# The genome sequence of the Cotton Bollworm moth, *Helicoverpa armigera *(Hübner, 1808)

**DOI:** 10.12688/wellcomeopenres.22882.1

**Published:** 2024-08-07

**Authors:** Peter W. H. Holland, University of Oxford and Wytham Woods Genome Acquisition Lab, Darwin Tree of Life Barcoding collective, Wellcome Sanger Institute Tree of Life Management, Samples and Laboratory team, Wellcome Sanger Institute Scientific Operations: Sequencing Operations, Wellcome Sanger Institute Tree of Life Core Informatics team, Tree of Life Core Informatics collective, Darwin Tree of Life Consortium

**Affiliations:** 1 Department of Biology, University of Oxford, Oxford, England, UK

**Keywords:** Helicoverpa armigera, Cotton Bollworm moth, genome sequence, chromosomal, Lepidoptera

## Abstract

We present a genome assembly from an adult female Cotton Bollworm moth, *Helicoverpa armigera* (Arthropoda; Insecta; Lepidoptera; Noctuidae). The genome sequence has a total length of 362.20 megabases. Most of the assembly is scaffolded into 32 chromosomal pseudomolecules, including the W and Z sex chromosomes. The mitochondrial genome has also been assembled and is 15.36 kilobases in length.

## Species taxonomy

Eukaryota; Opisthokonta; Metazoa; Eumetazoa; Bilateria; Protostomia; Ecdysozoa; Panarthropoda; Arthropoda; Mandibulata; Pancrustacea; Hexapoda; Insecta; Dicondylia; Pterygota; Neoptera; Endopterygota; Amphiesmenoptera; Lepidoptera; Glossata; Neolepidoptera; Heteroneura; Ditrysia; Obtectomera; Noctuoidea; Noctuidae; Heliothinae; *Helicoverpa*; *Helicoverpa armigera* (Hübner, 1808) (NCBI:txid29058).

## Background

The noctuid moth *Helicoverpa armigera* is one of the world’s most damaging and economically important agricultural insect pests. In Britain, where the moth is a sporadic and infrequent migrant, *H. armigera* is commonly known as the Scarce Bordered Straw; in much of the rest of world the moth is called the Cotton Bollworm or the Old World Bollworm. The larvae of *H. armigera* are polyphagous: over 180 host plants have been recorded, including maize, tomato, cotton and other key crops ( USDA, 2018). Typically, the larvae feed preferentially on fruits and flowers, but they will also eat leaves and shoots ( USDA, 2018). Larval development can be rapid, such that in temperate regions *H. armigera* has between two and five generations each year; in tropical regions the species may achieve up to 11 generations per year ( USDA, 2018).

The native range of *H. armigera* is thought to have encompassed southern Europe, North Africa, sub-Saharan Africa, Asia and Australia ( Hardwick, 1965; Nibouche *et al.*, 1998). In much of its original range the species is now a serious pest, with the larvae causing extensive damage to crops. The ability for long-distance flight, extreme polyphagy, rapid development and evolution of broad pesticide resistance all combine to give *H. armigera* the status of ‘megapest’ ( Tembrock *et al.*, 2019). Specimens of the adult moth recorded in Britain are thought to be adventitious immigrants from southern Europe, and the species has not become resident or become a crop pest.

As a result of accidental introductions by human activity, the species has recently spread to the Americas. *H. armigera* was recorded from several states across Brazil in 2013 ( Czepak *et al.*, 2013; Tay *et al.*, 2013), although analysis of museum specimens suggests the species reached Brazil several years earlier, at least by 2008 ( Sosa-Gómez *et al.*, 2016). Since its introduction, *H. armigera* has since become established as a major agricultural pest of cotton and soybean in Brazil ( Queiroz-Santos *et al.*, 2018), and has spread to neighbouring countries including Peru and Uruguay ( Castiglioni *et al.*, 2016). Annual crop losses in Brazil alone are estimated at over $1 billion per year, comparable to crop losses caused by the same pest in China ( Mastrangelo *et al.*, 2014). Mapping the spread of the species has been complicated by confusion with the closely related New World species *H. zea* ( Queiroz-Santos *et al.*, 2018) and through hybridisation between the two species ( Cordeiro *et al.*, 2020). *H. armigera* has been intercepted many times in imported plant material at border control checks into the United States, and some adults have been caught free-flying. Interception strategies have been effective and the species is not yet established as an invasive pest in the United States ( USDA, 2018).

In light of the economic importance of the species, several genome sequences for *H. armigera* have been obtained. For example, Pearce *et al*. (2017) determined a scaffold-level assembly from specimens collected in Australia. Zhang *et al*. (2022) reported a chromosome-level assembly from the SCD strain originally from Africa. Jin *et al*. (2023) published a chromosome-level assembly for a specimen from China, and used extensive resequencing to map the spread of *H. armigera* across the world. Here we report a complete genome sequence from a migratory specimen of *H. armigera* collected in Britain as part of the Darwin Tree of Life project. The genome sequence will be useful for comparative studies across the range of this cosmopolitan species and will give further insights into the biology of this economically important insect species.

## Genome sequence report

**Figure 1.**
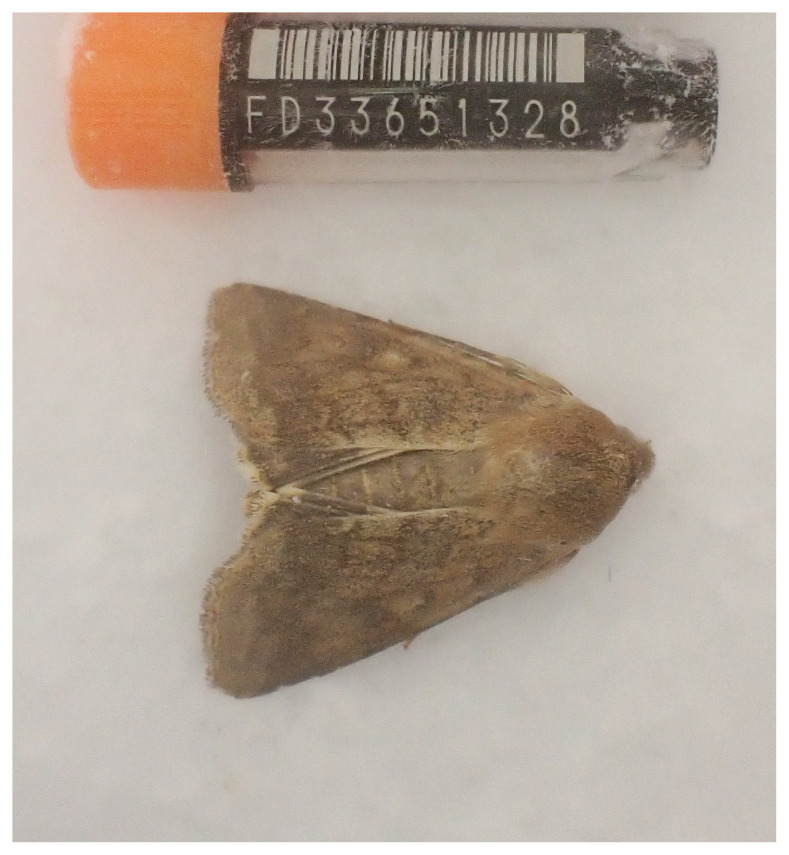
Photograph of the *Helicoverpa armigera* (ilHelArmi9) specimen used for genome sequencing.

The genome of an adult female *Helicoverpa armigera* ( Figure 1) was sequenced using Pacific Biosciences single-molecule HiFi long reads, generating a total of 30.42 Gb (gigabases) from 2.37 million reads, providing approximately 81-fold coverage. Primary assembly contigs were scaffolded with chromosome conformation Hi-C data, which produced 116.65 Gbp from 772.52 million reads, yielding an approximate coverage of 322-fold. Specimen and sequencing information is summarised in Table 1.

**Table 1.** Specimen and sequencing data for *Helicoverpa armigera*.

Project information			
**Study title**	*Helicoverpa armigera* (cotton bollworm)		
**Umbrella BioProject**	PRJEB66754		
**Species**	*Helicoverpa armigera*		
**BioSample**	SAMEA112775024		
**NCBI taxonomy ID**	29058		
Specimen information			
**Technology**	**ToLID**	**BioSample accession**	**Organism part**
**PacBio long read sequencing**	ilHelArmi9	SAMEA112775094	thorax
**Hi-C sequencing**	ilHelArmi9	SAMEA112775093	head
Sequencing information			
**Platform**	**Run accession**	**Read count**	**Base count (Gb)**
**Hi-C Illumina NovaSeq 6000**	ERR12102422	7.73e+08	116.65
**PacBio Sequel IIe**	ERR12102456	2.37e+06	30.42

**Figure 2.**
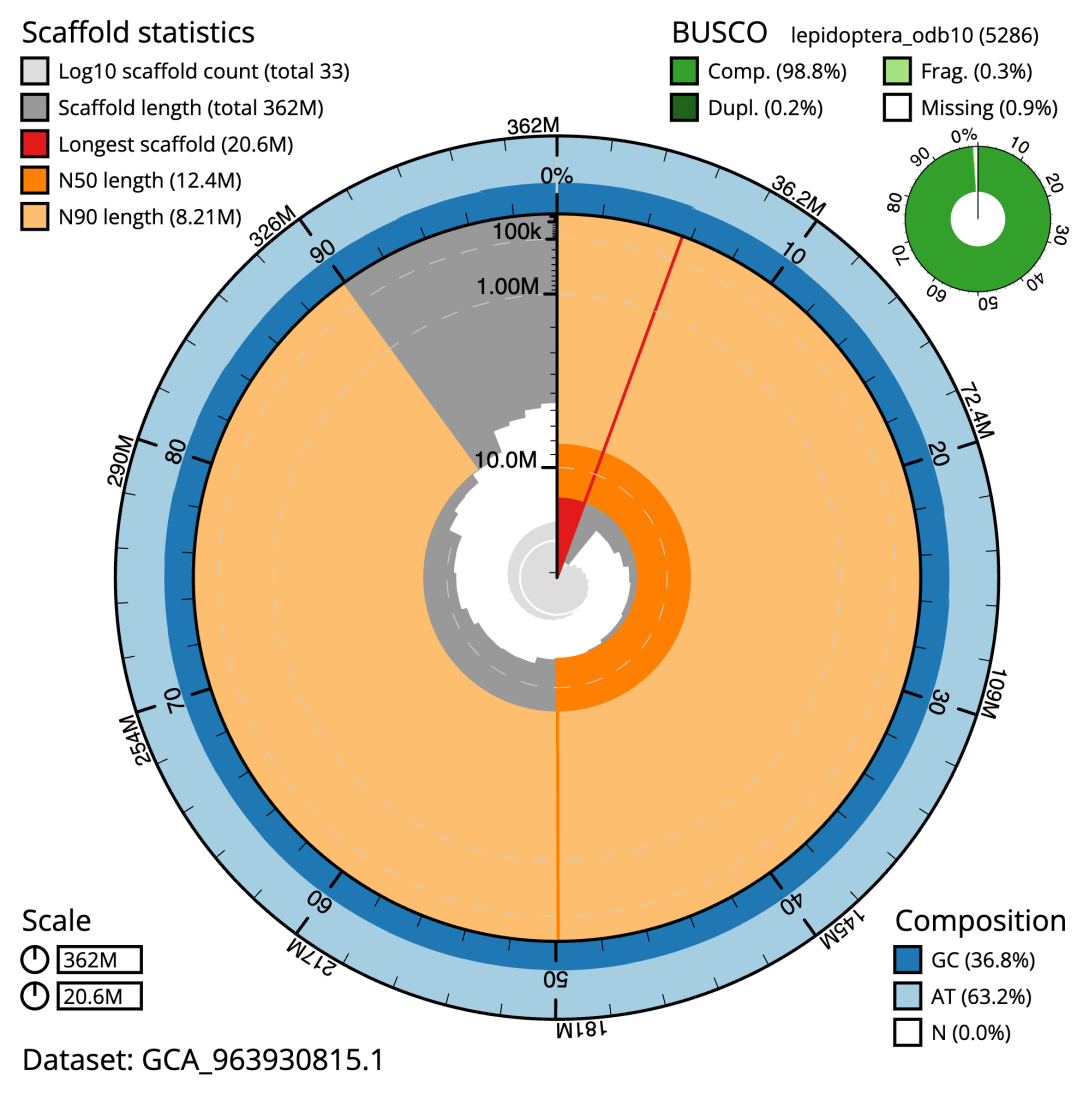
Genome assembly of *Helicoverpa armigera*, ilHelArmi9.1: metrics. The BlobToolKit snail plot shows N50 metrics and BUSCO gene completeness. The main plot is divided into 1,000 size-ordered bins around the circumference with each bin representing 0.1% of the 362,193,782 bp assembly. The distribution of scaffold lengths is shown in dark grey with the plot radius scaled to the longest scaffold present in the assembly (20,559,992 bp, shown in red). Orange and pale-orange arcs show the N50 and N90 scaffold lengths (12,359,365 and 8,208,105 bp), respectively. The pale grey spiral shows the cumulative scaffold count on a log scale with white scale lines showing successive orders of magnitude. The blue and pale-blue area around the outside of the plot shows the distribution of GC, AT and N percentages in the same bins as the inner plot. A summary of complete, fragmented, duplicated and missing BUSCO genes in the lepidoptera_odb10 set is shown in the top right. An interactive version of this figure is available at https://blobtoolkit.genomehubs.org/view/GCA_963930815.1/dataset/GCA_963930815.1/snail.

**Figure 3.**
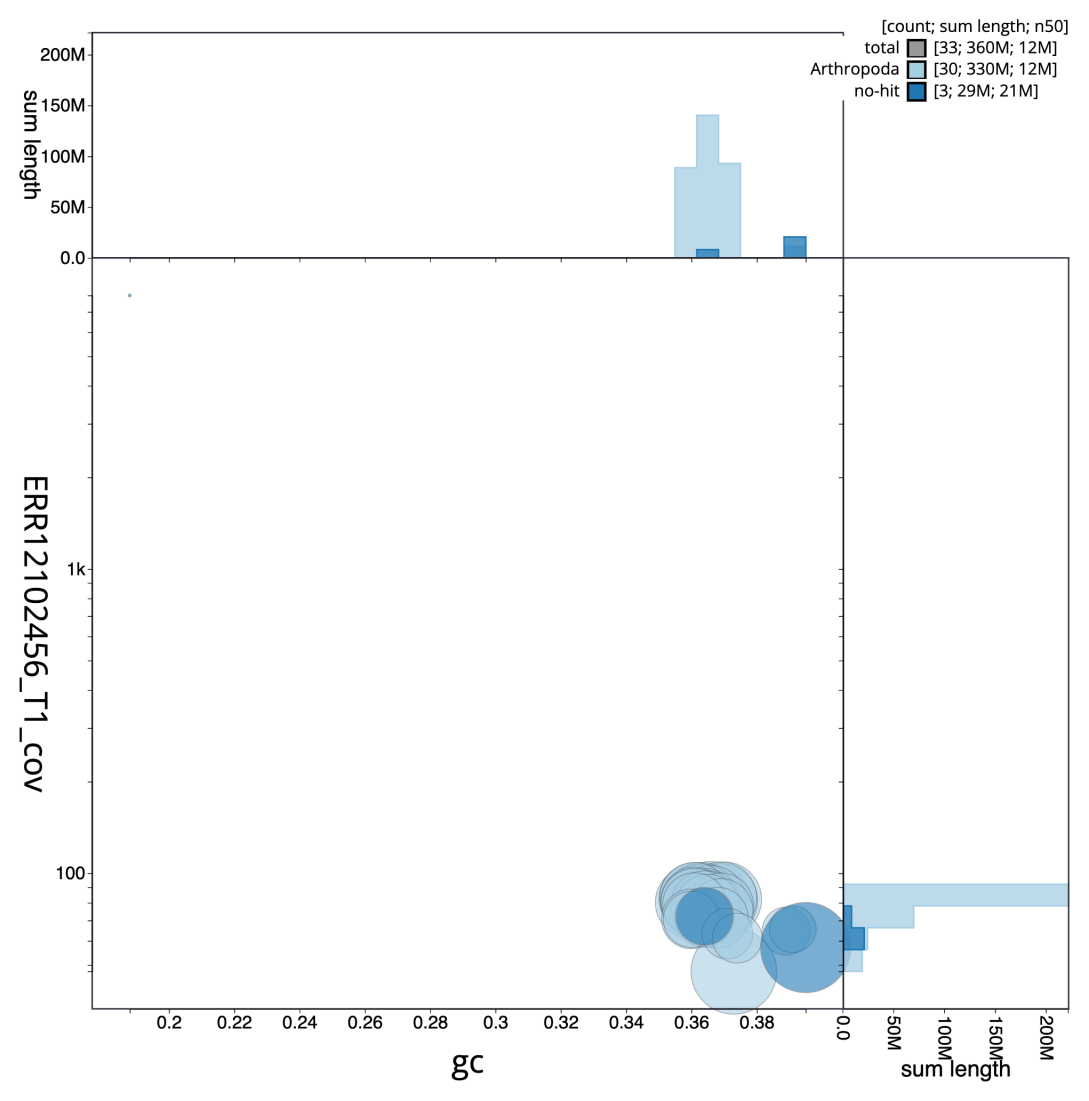
Genome assembly of *Helicoverpa armigera*, ilHelArmi9.1: BlobToolKit GC-coverage plot. Sequences are coloured by phylum. Circles are sized in proportion to sequence length. Histograms show the distribution of sequence length sum along each axis. An interactive version of this figure is available at https://blobtoolkit.genomehubs.org/view/GCA_963930815.1/dataset/GCA_963930815.1/blob.

**Figure 4.**
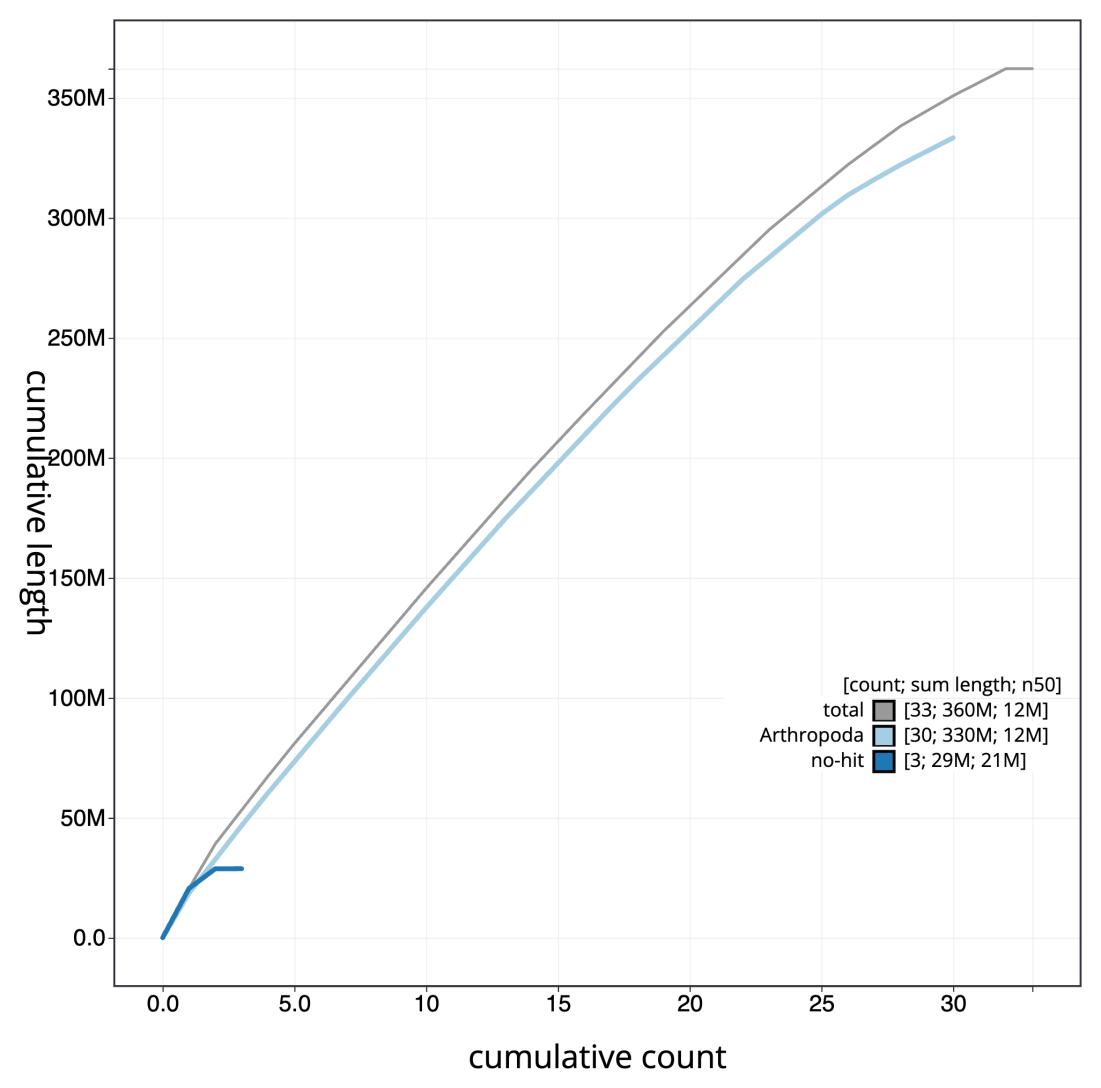
Genome assembly of *Helicoverpa armigera* ilHelArmi9.1: BlobToolKit cumulative sequence plot. The grey line shows cumulative length for all sequences. Coloured lines show cumulative lengths of sequences assigned to each phylum using the buscogenes taxrule. An interactive version of this figure is available at https://blobtoolkit.genomehubs.org/view/GCA_963930815.1/dataset/GCA_963930815.1/cumulative.

**Figure 5.**
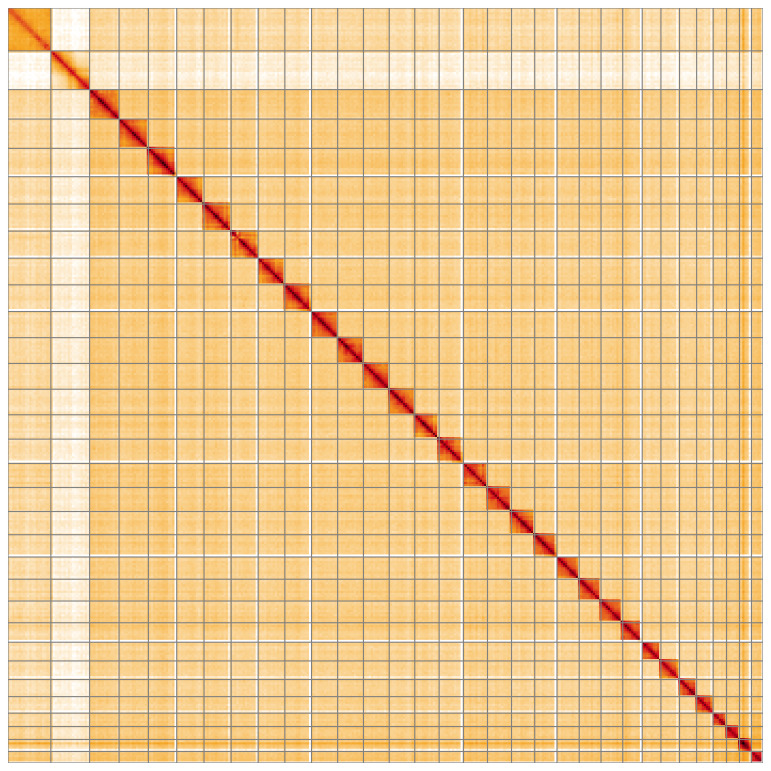
Genome assembly of *Helicoverpa armigera* ilHelArmi9.1: Hi-C contact map of the ilHelArmi9.1 assembly, visualised using HiGlass. Chromosomes are shown in order of size from left to right and top to bottom. An interactive version of this figure may be viewed at https://genome-note-higlass.tol.sanger.ac.uk/l/?d=C78zRt_BSJWdJwveADx4yg.

Manual assembly curation corrected three missing joins or mis-joins and one haplotypic duplication, reducing the assembly length by 26.67% and the scaffold number by 98.6%, and increasing the scaffold N50 by 11.0%. The final assembly has a total length of 362.20 Mb in 32 sequence scaffolds, with 16 gaps. The scaffold N50 is 12.4 Mb ( Table 2). The snail plot in Figure 2 provides a summary of the assembly statistics, while the distribution of assembly scaffolds on GC proportion and coverage is shown in Figure 3. The cumulative assembly plot in Figure 4 shows curves for subsets of scaffolds assigned to different phyla. Most (99.99%) of the assembly sequence was assigned to 32 chromosomal-level scaffolds, representing 30 autosomes and the W and Z sex chromosomes. Chromosome-scale scaffolds confirmed by the Hi-C data are named in order of size ( Figure 5; Table 3). While not fully phased, the assembly deposited is of one haplotype. Contigs corresponding to the second haplotype have also been deposited. The mitochondrial genome was also assembled and can be found as a contig within the multifasta file of the genome submission.

**Table 2.** Genome assembly data for *Helicoverpa armigera*, ilHelArmi9.1.

Genome assembly		
Assembly name	ilHelArmi9.1	
Assembly accession	GCA_963930815.1	
*Accession of alternate haplotype*	*GCA_963930805.1*	
Span (Mb)	362.20	
Number of contigs	49	
Contig N50 length (Mb)	11.2	
Number of scaffolds	32	
Scaffold N50 length (Mb)	12.4	
Longest scaffold (Mb)	20.56	
Assembly metrics *		*Benchmark*
Consensus quality (QV)	69.9	*≥ 50*
*k*-mer completeness	100.0%	*≥ 95%*
BUSCO **	C:98.7%[S:98.5%,D:0.2%], F:0.3%,M:1.0%,n:5,286	*C ≥ 95%*
Percentage of assembly mapped to chromosomes	99.99%	*≥ 95%*
Sex chromosomes	WZ	*localised homologous pairs*
Organelles	Mitochondrial genome: 15.36 kb	*complete single alleles*

* Assembly metric benchmarks are adapted from column VGP-2020 of “Table 1: Proposed standards and metrics for defining genome assembly quality” from Rhie *et al.* (2021). ** BUSCO scores based on the lepidoptera_odb10 BUSCO set using version 5.4.3. C = complete [S = single copy, D = duplicated], F = fragmented, M = missing, n = number of orthologues in comparison. A full set of BUSCO scores is available at https://blobtoolkit.genomehubs.org/view/Helicoverpa_armigera/dataset/GCA_963930815.1/busco.

**Table 3.** Chromosomal pseudomolecules in the genome assembly of *Helicoverpa armigera*, ilHelArmi9.

INSDC accession	Name	Length (Mb)	GC%
OZ005767.1	1	14.13	37.0
OZ005768.1	2	14.07	36.5
OZ005769.1	3	13.58	37.0
OZ005770.1	4	13.11	36.0
OZ005771.1	5	12.99	37.0
OZ005772.1	6	12.97	36.5
OZ005773.1	7	12.81	36.0
OZ005774.1	8	12.79	36.5
OZ005775.1	9	12.53	36.5
OZ005776.1	10	12.38	36.0
OZ005777.1	11	12.36	36.5
OZ005778.1	12	12.26	36.0
OZ005779.1	13	11.69	36.5
OZ005780.1	14	11.65	36.0
OZ005781.1	15	11.55	36.5
OZ005782.1	16	11.52	36.0
OZ005783.1	17	11.13	36.5
OZ005784.1	18	10.68	37.0
OZ005785.1	19	10.66	36.5
OZ005786.1	20	10.46	37.0
OZ005787.1	21	10.42	36.0
OZ005788.1	22	9.33	37.0
OZ005789.1	23	8.98	36.0
OZ005790.1	24	8.92	36.5
OZ005791.1	25	8.21	36.5
OZ005792.1	26	7.92	36.0
OZ005793.1	27	6.43	37.0
OZ005794.1	28	6.2	37.5
OZ005795.1	29	5.76	39.0
OZ005796.1	30	5.51	39.0
OZ005765.1	W	20.56	39.5
OZ005766.1	Z	18.59	37.5
OZ005797.1	MT	0.02	19.0

The estimated Quality Value (QV) of the final assembly is 69.9 with *k*-mer completeness of 100.0%, and the assembly has a BUSCO v5.4.3 completeness of 98.7% (single = 98.5%, duplicated = 0.2%), using the lepidoptera_odb10 reference set ( *n* = 5,286).

Metadata for specimens, BOLD barcode results, spectra estimates, sequencing runs, contaminants and pre-curation assembly statistics are given at https://links.tol.sanger.ac.uk/species/29058.

## Methods

### Sample acquisition

An adult *Helicoverpa armigera* (specimen ID Ox003089, ToLID ilHelArmi9) was collected from Wallingford, Berkshire, UK (latitude 51.6, longitude –1.14) on 2022-10-22 using a light trap. The specimen was collected and identified by Peter Holland (University of Oxford) and preserved on dry ice.

The initial species identification was verified by an additional DNA barcoding process according to the framework developed by Twyford *et al*. (2024). A small sample was dissected from the specimens and stored in ethanol, while the remaining parts of the specimen were shipped on dry ice to the Wellcome Sanger Institute (WSI). The tissue was lysed, the COI marker region was amplified by PCR, and amplicons were sequenced and compared to the BOLD database, confirming the species identification ( Crowley *et al.*, 2023). Following whole genome sequence generation, the relevant DNA barcode region was also used alongside the initial barcoding data for sample tracking at the WSI ( Twyford *et al.*, 2024). The standard operating procedures for Darwin Tree of Life barcoding have been deposited on protocols.io ( Beasley *et al.*, 2023).

### Nucleic acid extraction

The workflow for high molecular weight (HMW) DNA extraction at the WSI Tree of Life Core Laboratory includes a sequence of core procedures: sample preparation and homogenisation, DNA extraction, fragmentation and purification. Detailed protocols are publicly available on protocols.io ( Denton *et al.*, 2023b).

The sample was prepared at the Tree of Life Core Laboratory. The ilHelArmi9 sample was weighed and dissected on dry ice ( Jay *et al.*, 2023) and tissue from the thorax was homogenised using a PowerMasher II tissue disruptor ( Denton *et al.*, 2023a).

HMW DNA was extracted in the WSI Scientific Operations core using the Automated MagAttract v2 protocol ( Oatley *et al.*, 2023). The DNA was sheared into an average fragment size of 12–20 kb in a Megaruptor 3 system ( Bates *et al.*, 2023). Sheared DNA was purified by solid-phase reversible immobilisation, using AMPure PB beads to sample to eliminate shorter fragments and concentrate the DNA ( Strickland *et al.*, 2023). The concentration of the sheared and purified DNA was assessed using a Nanodrop spectrophotometer and Qubit Fluorometer using the Qubit dsDNA High Sensitivity Assay kit. Fragment size distribution was evaluated by running the sample on the FemtoPulse system.

### Sequencing

Pacific Biosciences HiFi circular consensus DNA sequencing libraries were constructed according to the manufacturers’ instructions. DNA sequencing was performed by the Scientific Operations core at the WSI on a Pacific Biosciences Sequel IIe instrument. Hi-C data were also generated from head tissue of ilHelArmi9 using the Arima-HiC v2 kit. The Hi-C sequencing was performed using paired-end sequencing with a read length of 150 bp on the Illumina NovaSeq 6000 instrument.

### Genome assembly, curation and evaluation

***Assembly***

The original assembly of HiFi reads was performed using Hifiasm ( Cheng *et al.*, 2021) with the --primary option. Haplotypic duplications were identified and removed using purge_dups ( Guan *et al.*, 2020). Hi-C reads were mapped to primary contigs with bwa-mem2 ( Vasimuddin *et al.*, 2019). The primary contigs were scaffolded using the provided Hi-C data ( Rao *et al.*, 2014) in YaHS ( Zhou *et al.*, 2023), using the --break option. The scaffolded assemblies were evaluated using Gfastats ( Formenti *et al.*, 2022), BUSCO ( Manni *et al.*, 2021) and MERQURY.FK ( Rhie *et al.*, 2020).

The mitochondrial genome was assembled using MitoHiFi ( Uliano-Silva *et al.*, 2023), which runs MitoFinder ( Allio *et al.*, 2020) and uses these annotations to select the final mitochondrial contig and to ensure the general quality of the sequence.

***Assembly curation***

The assembly was decontaminated using the Assembly Screen for Cobionts and Contaminants (ASCC) pipeline (article in preparation). Flat files and maps used in curation were generated in TreeVal ( Pointon *et al.*, 2023). Manual curation was primarily conducted using PretextView ( Harry, 2022), with additional insights provided by JBrowse2 ( Diesh *et al.*, 2023) and HiGlass ( Kerpedjiev *et al.*, 2018). Scaffolds were visually inspected and corrected as described by Howe *et al*. (2021). Any identified contamination, missed joins, and mis-joins were corrected, and duplicate sequences were tagged and removed. The sex chromosomes were identified by read coverage statistics. The entire process is documented at https://gitlab.com/wtsi-grit/rapid-curation (article in preparation).

***Evaluation of the final assembly***

The final assembly was post-processed and evaluated with the three Nextflow ( Di Tommaso *et al.*, 2017) DSL2 pipelines “sanger-tol/readmapping” ( Surana *et al.*, 2023a), “sanger-tol/genomenote” ( Surana *et al.*, 2023b), and “sanger-tol/blobtoolkit” ( Muffato *et al.*, 2024). The pipeline sanger-tol/readmapping aligns the Hi-C reads with bwa-mem2 ( Vasimuddin *et al.*, 2019) and combines the alignment files with SAMtools ( Danecek *et al.*, 2021). The sanger-tol/genomenote pipeline transforms the Hi-C alignments into a contact map with BEDTools ( Quinlan & Hall, 2010) and the Cooler tool suite ( Abdennur & Mirny, 2020), which is then visualised with HiGlass ( Kerpedjiev *et al.*, 2018). It also provides statistics about the assembly with the NCBI datasets ( Sayers *et al.*, 2024) report, computes *k*-mer completeness and QV consensus quality values with FastK and MERQURY.FK, and a completeness assessment with BUSCO ( Manni *et al.*, 2021).

The sanger-tol/blobtoolkit pipeline is a Nextflow port of the previous Snakemake Blobtoolkit pipeline ( Challis *et al.*, 2020). It aligns the PacBio reads with SAMtools and minimap2 ( Li, 2018) and generates coverage tracks for regions of fixed size. In parallel, it queries the GoaT database ( Challis *et al.*, 2023) to identify all matching BUSCO lineages to run BUSCO ( Manni *et al.*, 2021). For the three domain-level BUSCO lineage, the pipeline aligns the BUSCO genes to the Uniprot Reference Proteomes database ( Bateman *et al.*, 2023) with DIAMOND ( Buchfink *et al.*, 2021) blastp. The genome is also split into chunks according to the density of the BUSCO genes from the closest taxonomically lineage, and each chunk is aligned to the Uniprot Reference Proteomes database with DIAMOND blastx. Genome sequences that have no hit are then chunked with seqtk and aligned to the NT database with blastn ( Altschul *et al.*, 1990). All those outputs are combined with the blobtools suite into a blobdir for visualisation.

The genome assembly and evaluation pipelines were developed using the nf-core tooling ( Ewels *et al.*, 2020), use MultiQC ( Ewels *et al.*, 2016), and make extensive use of the Conda package manager, the Bioconda initiative ( Grüning *et al.*, 2018), the Biocontainers infrastructure ( da Veiga Leprevost *et al.*, 2017), and the Docker ( Merkel, 2014) and Singularity ( Kurtzer *et al.*, 2017) containerisation solutions.

Table 4 contains a list of relevant software tool versions and sources.

**Table 4.** Software tools: versions and sources.

Software tool	Version	Source
BEDTools	2.30.0	https://github.com/arq5x/bedtools2
BLAST	2.14.0	ftp://ftp.ncbi.nlm.nih.gov/blast/executables/blast+/
BlobToolKit	4.3.7	https://github.com/blobtoolkit/blobtoolkit
BUSCO	5.4.3 and 5.5.0	https://gitlab.com/ezlab/busco
bwa-mem2	2.2.1	https://github.com/bwa-mem2/bwa-mem2
Cooler	0.8.11	https://github.com/open2c/cooler
DIAMOND	2.1.8	https://github.com/bbuchfink/diamond
fasta_windows	0.2.4	https://github.com/tolkit/fasta_windows
FastK	427104ea91c78c3b8b8b49f1a7d6bbeaa869ba1c	https://github.com/thegenemyers/FASTK
Gfastats	1.3.6	https://github.com/vgl-hub/gfastats
GoaT CLI	0.2.5	https://github.com/genomehubs/goat-cli
Hifiasm	0.19.5-r587	https://github.com/chhylp123/hifiasm
HiGlass	44086069ee7d4d3f6f3f0012569789ec138f42b84 aa44357826c0b6753eb28de	https://github.com/higlass/higlass
Merqury.FK	d00d98157618f4e8d1a9190026b19b471055b22e	https://github.com/thegenemyers/MERQURY.FK
MitoHiFi	3	https://github.com/marcelauliano/MitoHiFi
MultiQC	1.14, 1.17, and 1.18	https://github.com/MultiQC/MultiQC
NCBI Datasets	15.12.0	https://github.com/ncbi/datasets
Nextflow	23.04.0-5857	https://github.com/nextflow-io/nextflow
PretextView	0.2	https://github.com/sanger-tol/PretextView
purge_dups	1.2.5	https://github.com/dfguan/purge_dups
samtools	1.16.1, 1.17, and 1.18	https://github.com/samtools/samtools
sanger-tol/ascc	-	https://github.com/sanger-tol/ascc
sanger-tol/genomenote	1.1.1	https://github.com/sanger-tol/genomenote
sanger-tol/readmapping	1.2.1	https://github.com/sanger-tol/readmapping
Seqtk	1.3	https://github.com/lh3/seqtk
Singularity	3.9.0	https://github.com/sylabs/singularity
TreeVal	1.0.0	https://github.com/sanger-tol/treeval
YaHS	1.2a.2	https://github.com/c-zhou/yahs

### Wellcome Sanger Institute – Legal and Governance

The materials that have contributed to this genome note have been supplied by a Darwin Tree of Life Partner. The submission of materials by a Darwin Tree of Life Partner is subject to the **‘Darwin Tree of Life Project Sampling Code of Practice’**, which can be found in full on the Darwin Tree of Life website here. By agreeing with and signing up to the Sampling Code of Practice, the Darwin Tree of Life Partner agrees they will meet the legal and ethical requirements and standards set out within this document in respect of all samples acquired for, and supplied to, the Darwin Tree of Life Project.

Further, the Wellcome Sanger Institute employs a process whereby due diligence is carried out proportionate to the nature of the materials themselves, and the circumstances under which they have been/are to be collected and provided for use. The purpose of this is to address and mitigate any potential legal and/or ethical implications of receipt and use of the materials as part of the research project, and to ensure that in doing so we align with best practice wherever possible. The overarching areas of consideration are:

- Ethical review of provenance and sourcing of the material
- Legality of collection, transfer and use (national and international)

Each transfer of samples is further undertaken according to a Research Collaboration Agreement or Material Transfer Agreement entered into by the Darwin Tree of Life Partner, Genome Research Limited (operating as the Wellcome Sanger Institute), and in some circumstances other Darwin Tree of Life collaborators.

## Data Availability

European Nucleotide Archive: *Helicoverpa armigera* (cotton bollworm). Accession number PRJEB66754; https://identifiers.org/ena.embl/PRJEB66754 ( Wellcome Sanger Institute, 2024). The genome sequence is released openly for reuse. The *Helicoverpa armigera* genome sequencing initiative is part of the Darwin Tree of Life (DToL) project. All raw sequence data and the assembly have been deposited in INSDC databases. The genome will be annotated using available RNA-Seq data and presented through the Ensembl pipeline at the European Bioinformatics Institute. Raw data and assembly accession identifiers are reported in Table 1 and Table 2.

## Author information

Members of the University of Oxford and Wytham Woods Genome Acquisition Lab are listed here: https://doi.org/10.5281/zenodo.12157525.

Members of the Darwin Tree of Life Barcoding collective are listed here: https://doi.org/10.5281/zenodo.12158331

Members of the Wellcome Sanger Institute Tree of Life Management, Samples and Laboratory team are listed here: https://doi.org/10.5281/zenodo.12162482.

Members of Wellcome Sanger Institute Scientific Operations: Sequencing Operations are listed here: https://doi.org/10.5281/zenodo.12165051.

Members of the Wellcome Sanger Institute Tree of Life Core Informatics team are listed here: https://doi.org/10.5281/zenodo.12160324.

Members of the Tree of Life Core Informatics collective are listed here: https://doi.org/10.5281/zenodo.12205391.

Members of the Darwin Tree of Life Consortium are listed here: https://doi.org/10.5281/zenodo.4783558.
