# Supplementary material for: The genome sequence of the Cotton Bollworm moth, Helicoverpa armigera (Hübner, 1808)
Source: Wellcome Open Res. 2024 Aug 7;9:428. [Version 1] doi: 10.12688/wellcomeopenres.22882.1 (PMC11462122; doi:10.12688/wellcomeopenres.22882.1)
Supplement: Peer review history

# Reviewer report: Reviewer response for version 1

**DOI:** 10.21956/wellcomeopenres.25197.r101335

**Published:** 2024-10-19

**Authors:** Lino Ometto

**Affiliations:** 1 University of Pavia, Pavia, Italy

This manuscript by PWH Holand provides a comprehensive description of the methods and results of a genome assembly from an adult female Cotton Bollworm moth, *Helicoverpa armigera.*

**I only have few very minor suggestions that the author may consider to include in the manuscript**:

  

- Assembly section: rather than saying that “The scaffolded assemblies were evaluated” I would write “The quality and completeness of the scaffolded assemblies were evaluated”
- Would it make sense to add chromosome number (and especially identify W and Z) in Figure 5?
- The sequenced specimen was a female, but under Sample acquisition is not clear whether this was a deliberate choice (i.e. to obtain both W and Z), since it is not mentioned whether the moth was sexed before being processed.
- It would be interesting to provide a brief comparison to the other currently available genomes of the same species (https://www.ncbi.nlm.nih.gov/datasets/genome/?taxon=29058), e.g. difference in chromosome number, size and completeness, to highlight the better quality of this assembled genome.

Are sufficient details of methods and materials provided to allow replication by others?

Yes

Is the rationale for creating the dataset(s) clearly described?

Yes

Are the datasets clearly presented in a useable and accessible format?

Yes

Are the protocols appropriate and is the work technically sound?

Yes

Reviewer Expertise:

Population genetics in insects

I confirm that I have read this submission and believe that I have an appropriate level of expertise to confirm that it is of an acceptable scientific standard.

# Reviewer report: Reviewer response for version 1

**DOI:** 10.21956/wellcomeopenres.25197.r101329

**Published:** 2024-10-08

**Authors:** Thomas Simonsen

**Affiliations:** 1 Natural History Museum Aarhus, Aarhus, Denmark

I am not 100% into the technical side of whole genome sequencing, but the manuscript seems flawless to me. The only, and admittedly very small, request I can think of is that it would be good to know how the specimen was killed. Was it frozen alive, or was it killed using a standard method and then transferred to dry ice. This is somewhat relevant for others who intend to collect material for similar purpose.

Are sufficient details of methods and materials provided to allow replication by others?

Yes

Is the rationale for creating the dataset(s) clearly described?

Yes

Are the datasets clearly presented in a useable and accessible format?

Yes

Are the protocols appropriate and is the work technically sound?

Yes

Reviewer Expertise:

Insect diversity

I confirm that I have read this submission and believe that I have an appropriate level of expertise to confirm that it is of an acceptable scientific standard.

# Reviewer report: Reviewer response for version 1

**DOI:** 10.21956/wellcomeopenres.25197.r97592

**Published:** 2024-09-12

**Authors:** Boopathi N Manikanda

**Affiliations:** 1 Department of Plant Biotechnology, CPMB&B, Tamil Nadu Agricultural University, Coimbatore, Tamil Nadu, India

**Materials and Methods:**

 While nucleic acid extraction and processing the short sheared DNA fragments were eliminated and DNA were concentrated. What kind of measures were taken to avoid the errors during assembly and annotation that occur due to the loss of highly repetitive sequences (which may have short repeat motif, for example)? 

 Such effort may be highlighted additionally under this subheading. 

**Dataset:**

 A final conclusion on how this data set differ from other related dataset of *Helicoverpa armigera *and what are the unique barcoding regions that can be used to differentiate such species in cotton with other crop specific *Helicoverpa armigera*?

Are sufficient details of methods and materials provided to allow replication by others?

Partly

Is the rationale for creating the dataset(s) clearly described?

Yes

Are the datasets clearly presented in a useable and accessible format?

Yes

Are the protocols appropriate and is the work technically sound?

Yes

Reviewer Expertise:

Genomics assisted breeding in Cotton, Moringa, Tomato, Cucumber and Tea

I confirm that I have read this submission and believe that I have an appropriate level of expertise to confirm that it is of an acceptable scientific standard.
